# A novel Longshi Scale measured activity of daily living disability in elderly patients affected by neurological diseases: a multi-center cross-sectional study in China

**DOI:** 10.1186/s12877-021-02296-6

**Published:** 2021-06-05

**Authors:** Jingpu Zhao, Xiangxiang Liu, Li Wan, Yan Gao, Meiling Huang, Fubing Zha, Jianjun Long, Dongxia Li, Guohui Nie, Yulong Wang

**Affiliations:** Department of Rehabilitation, Shenzhen Second People’s Hospital, The First Affiliated Hospital of Shenzhen University, Shenzhen, China

**Keywords:** Longshi Scale, Barthel Index, Activities of Daily Life, Older Adults

## Abstract

**Background:**

Ability in the activities of daily life is often impaired in the older adults with a neurological disease. The Barthel Index is an instrument used worldwide to assess such ability. The Longshi Scale is a picture-based alternative, but its effectiveness has not been evaluated with older adult subjects. This study was to determine whether the Longshi Scale can effectively quantify the ability of older adults in the activities of daily living by comparing its ratings with those using the Barthel Index.

**Methods:**

A multi-center cross-sectional study was conducted among patients over 65 years. A total of 2438 patients were divided into three groups, including bedridden, domestic, or community group based on their ability to go out of bed, move outdoors, and return indoors. Their ability in the activities of daily living among three groups was evaluated using both the Longshi Scale and the Barthel Index, and the results were compared.

**Results:**

There was a significant difference in the average Barthel Index scores of three groups classified using the Longshi Scale. The average Longshi Scale scores also showed significant differences between the four groups classified using the Barthel Index. Spearman correlation coefficients showed strong correlation(>0.83) between the Longshi Scale and Barthel Index scores.

**Conclusions:**

The Longshi Scale can efficiently distinguish the ability in the activities of daily living of people with a neurological disease. Its rating correlate well with those using the Barthel Index.

## Background

At the end of 2019 the total population of mainland China aged over 60 totaled more than 250 million, or about 18.1 % of the total population [1]. Older adults often have structural decline and degraded physiological functioning which may impair their ability to perform common activities of daily living (ADL) [2]. More seriously, loss of ADL ability may induce a poor quality of life to go with increased medical needs with their economic burden on the family and the healthcare system [3–6]. Therefore, it is important to evaluate the ADL disability in clinical practice, and the social security system demands that as well [7–9].

Katz [10] and others have proposed the ADL as a context to evaluating the health and functional status of older adults, that view has gradually been adopted. Several instruments have been developed for the purpose. The Barthel Index (BI) [11] is an instrument recommended by the Royal College of Physicians for routine use in the assessment of the ADL ability of older people [12, 13]. The index assesses ten daily activities using ordinal scales. The original BI was scored in steps of five points to give a maximum total score of 100. Functional Independence Measures have also been validated for evaluating the older adults [14]. They cover 18 items, with a total score ranging from 18 to 126. But using them properly requires professional training and is time-consuming. As an alternative, the Japanese have developed a Functional Independence and Difficulty Scale with 14 items, but it has been validated only with a Japanese population [15].

**Fig. 1:**
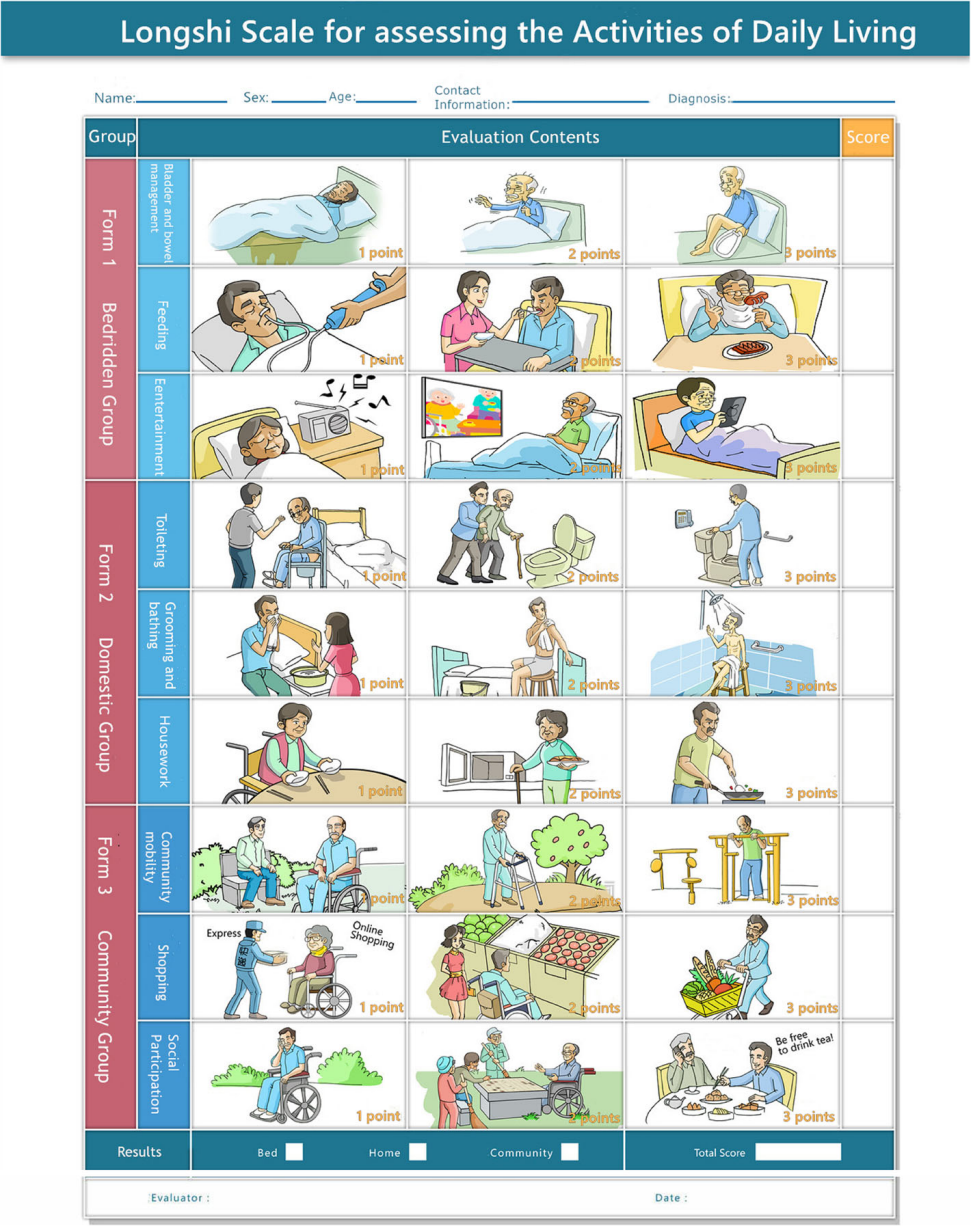
Longshi Scale for categories for assessing ability in the activities of daily living (owned by Corresponding author Yulong Wang)

**Fig. 2:**
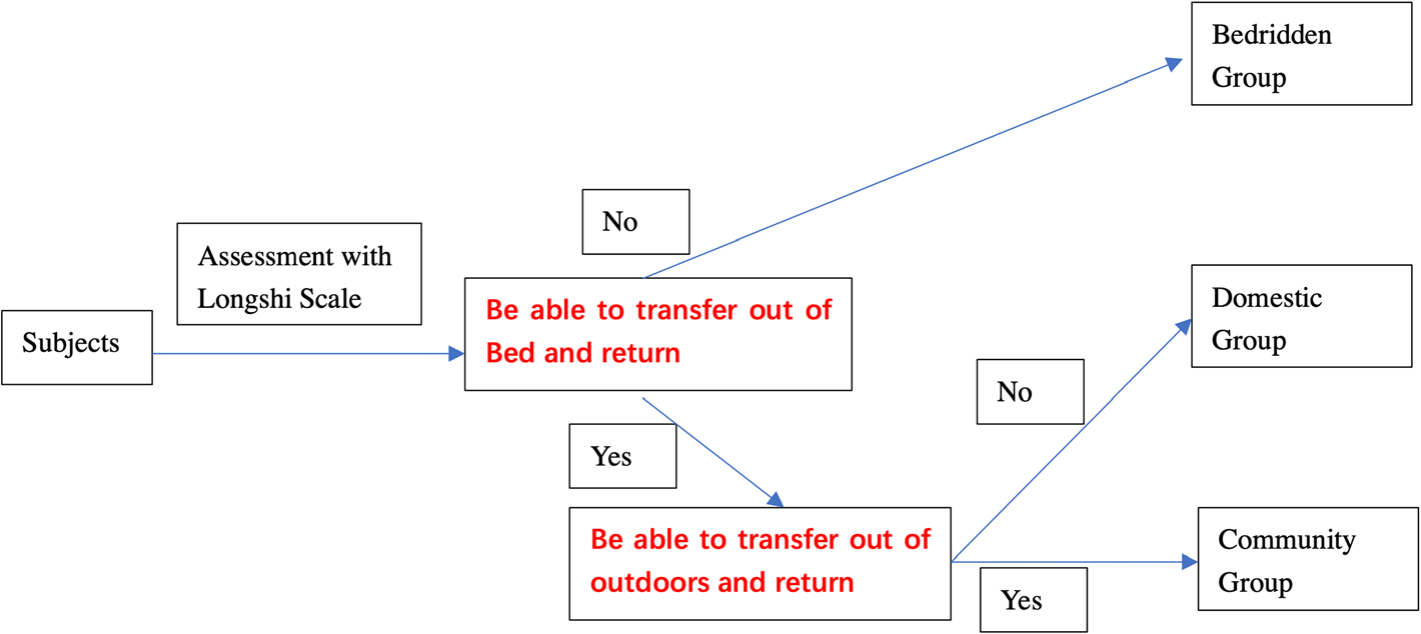
Flow chart of assessment using Longshi Scale

The Longshi Scale is a novel pictorial instrument, with good inter-rater and intra-rater reliability(Intraclass Correlation Coefficient, ICC>0.8) developed to assess disability associated with neuromuscular and musculoskeletal conditions [16]. A subject is first evaluated to assign them to the bedridden group, domestic group or community group (Fig. 1). The patient is then evaluated using a form to their particular group (Fig. 2). Each form consists of 3 items. Each item is assessed using three pictures by a professional corresponding to three levels on a Likert-type scale: 1 for maximum to fully dependent, 2 for partially independent, and 3 for maximum to fully independent. However, the Longshi Scale has not yet been validated for use with older people, especially those disabled by a medical condition.

The aim of this research was to document the extent to which the Longshi Scale can distinguish among levels of ADL ability with elderly disabled persons. Its ratings of elderly patients affected by neurological disease were compared with those of experienced professionals using the BI.

## Methods

### Study design and setting

This multi-center, cross-sectional study was conducted between October 2019 and April 2020 by 23 rehabilitation therapists from nine hospitals of Kunming, Chengdu, Shanghai and Shenzhen around China.

### Sample size calculation

An informal sample size calculation was performed. It was expected that the point estimate of proportions varies widely, therefore a prevalence of 0.4 of neurological diseases in the older adults (female, 50 %; male, 30 %) was assumed [17]. To measure this proportion with a 95 % confidence interval (CI) width of ± 0.05, 369 subjects were regarded as sufficient. In this study, 2438 older people with neurological diseases were included, which could meet the sample size.

### Participants

A total of 2438 inpatients in the rehabilitation medicine departments participated. For the purposes of this study, only neurological diseases patients (current diagnosed) were selected. The inclusion criteria were subjects who were age ≥ 65 years, a first-onset neurological condition confirmed by CT or MRI with a duration between 3 and 12 months, and with stable vital signs. The conditions represented were stroke, post-traumatic brain injury syndrome, brain tumor, spinal injury and other disorders. Patients who had diagnosed with mental illness or cognitive dysfunction were exclude. All the participants’ cognitive status had assessed by a nurse using Mini-mental State Examination (MMSE) before formal evaluation. In this study, we excluded the patients with mental illness or cognitive dysfunction(MMSE<27),because the Longshi Scale is a pictorial scale, and patients with MMSE<27 were unavailable to undergo Longshi Scale assessment. Additionally, patients participating in any other clinical study were also excluded.

### Measurement

The BI and Longshi Scale used in the study have no authorization restrictions, which can be obtained and used by the researchers freely. The BI [18] ratings of an individual’s performance of the 10 ADL tasks are based on the amount of physical assistance required to perform the task successfully. The BI ratings are in increments of 5 points (Table 1). The total scores range from 0 to 100. Scores for most items range from 0 to 10, with 0 being unable to perform, 5 assistance required, and 10 completely capable. Two items have a score range of 0 to 5, with 0 being unable and 5 complete ability. Two additional items score 0 to 15, with 0 being unable, 10 with assistance, and 15 fully capable. Based on previous studies, this study’s participants were classified as severe(0–20), moderate(21–60), mild(61–90), and no disability(91–100) using the BI [19].

**Table 1:** Bather Index scores

Items	Unable to perform the task	Needs assistance	Fully independent
Feeding	0	5	10
Bathing self	0	0	5
Personal hygiene	0	0	5
Dressing	0	5	10
Bowel control	0	5	10
Bladder control	0	5	10
Toilet	0	5	10
Chair/bed transfers	0	5–10	15
Ambulation	0	5–10	15
Wheelchair^a^	0	0	5
Stair climbing	0	5	10
Range	0		100

^a^Score only if unable to walk

A total BI score of 0–20 suggests total dependence, 21–60 severe dependence, 61–90 moderate dependence and 91–100 slight dependence. A score of 100 indicates that the patient can be independent of assistance from others in daily life

With all three groups (bedridden, domestic and community), the Longshi Scale assesses them using three pictorial items. The three-point Likert-type scales add up to a total possible score of 9 (with 3 indicating minimum independence and 9 indicating maximum independence) [15]. The participants were first assessed and classified into the bedridden, domestic, or community group based on their ability to get out of the bed, move outdoors and return indoors. (See the flow chart in Fig. 2). The bedridden group was defined as individuals could not get out of the bed independently, and the three items, including bladder and bowel management, feeding capability, and leisure activity, were assessed by professional evaluators based on caregiver-reported. Domestic group (domestic ambulator) was defined as individuals who could get out of bed but could not go outside independently with or without assistance (device or personal). Toileting, grooming, and housework were evaluated by professional evaluators based on caregiver- or patient-reported. Community group (also called community ambulator) was defined as individuals were capable of going outdoors independently with or without the help of assistive devices [15]. Community mobility, shopping and social participation were finally evaluated by professional evaluators based on caregiver- or patients-reported. (See the flow chart in Fig. 2).

### Data collection

Questionnaires were used to collect sociodemographic data about the subjects and about their lifestyles. The clinical information came from their medical records. ADL ability was assessed by a registered physician, nurses or therapist using the Quicker Recovery Line (QRL) platform, a rehabilitation evaluation and management system. Firstly, a professional logged in the QRL Application, and asked patients to signed informed consent. Secondly, the Longshi Scale and BI were pushed randomly based on QRL system. Lastly, the professional orderly used the pushed-scale to assess patients’ ADL. Both Longshi Scale and BI were evaluated by interviewing the patients or their caregivers face-to-face. All of the data were recorded on electronic forms and submitted to QRL online system. Missing information was completed by requesting that the practitioner involved re-interview the participant.

### Data analysis

Statistics describing the demographic characteristics and clinical information were compiled as numbers and percentages. The demographic characteristics included age, gender, occupation (civil servant, work, farmer and others), marital status(married or single including widowed), annual household income(<¥50,000; ¥50,000-100,000; ¥100,000-150,000 and >¥150,000),education level(primary or less, middle school, high school, college and higher) and diagnosis(cerebral hemorrhage, cerebral infarction, spinal and osteoarticular diseases, and others). All except the gender and diagnosis were self-reported and unverified. Concurrent chronic diseases including hypertension, diabetes, hyperlipidemia, and heart disease were also recorded in some cases.

All clinical and demographic variables of patients were performed with descriptive analyses. Means and standard deviations [SD] were computed for the continuous variables, while frequencies (proportions) were computed for categorical variables. Comparisons of the BI scores between the three Longshi Scale groups were made using one-way analysis of variance, as were the differences in Longshi Scale scores among the groups based on the BI results. Spearman correlation coefficients were computed to evaluate the relationships between the Longshi Scale scores and the BI scores. All of the analyses were conducted using version 21.0 of the SPSS software. A *p*-value of less than 0.05 was considered statistical significance, and all of the tests were two tailed.

## Results

There were 5944 inpatients with neurological diseases enrolled after preliminary screening. Given the inclusion/exclusion criteria, a total of 2438 subjects include in the study. There were 959 in the bedridden category, 644 considered domestic and 835 in the community group. More details of the three groups are shown in Table 2.

**Table 2:** Demographic and clinical characteristics of the subjects

Variables	Bedridden group(*n* = 959)	Domestic group(*n* = 644)	Community group(*n* = 835)	Total(*n* = 2438)
**Female, n(%)**	475(49.5)	299(46.4)	421(50.4)	1195(49.0)
**Age (Mean ± SD)**	78.40 ± 8.10	75.55 ± 7.09	71.78 ± 5.75	75.38 ± 7.64
**Profession**				
Civil servant^a^, n(%)	91(9.5)	57(8.9)	38(4.6)	186(7.6)
Worker^b^, n(%)	354(36.9)	141(21.9)	150(18.0)	645(26.5)
Farmer^c^, n(%)	232(24.2)	308(47.8)	546(65.4)	1086(44.5)
Others, n(%)	282(29.4)	138(21.4)	101(12.1)	521(21.3)
**Marital status**				
Single, n(%)	3(0.3)	7(1.1)	3(0.4)	13(0.5)
Married, n(%)	792(82.6)	565(87.7)	795(95.2)	2152(88.3)
Divorced, n(%)	8(0.8)	3(0.5)	5(0.6)	16(0.7)
Widowed, n(%)	156(16.3)	69(10.7)	32(3.8)	257(10.5)
**Education level**				
Primary School or below, n(%)	331(34.5)	346(53.7)	588(70.4)	1265(51.9)
Middle school, n(%)	245(25.5)	117(18.2)	122(14.6)	484(19.9)
High school	248(25.9)	100(15.5)	71(8.5)	419(17.2)
College or above, n(%)	99(10.3)	62(9.6)	42(5.0)	203(8.4)
Others, n(%)	36(3.8)	19(3.0)	12(1.4)	67(2.7)
**Family income**				
< 50,000 yuan, n(%)	265(27.6)	313(48.6)	528(63.2)	1106(45.4)
50,000-100,000 yuan, n(%)	332(34.6)	171(26.6)	188(22.5)	691(28.3)
100,000-150,000 yuan, n(%)	237(24.7)	111(17.2)	77(9.2)	425(17.4)
150,000-200,000 yuan, n(%)	70(7.3)	32(5.0)	26(3.1)	128(5.3)
> 200,000 yuan, n (%)	55(5.7)	17(2.6)	16(1.9)	88(3.6)
**Diagnosis**				
Cerebral hemorrhage, n(%)	139(14.5)	41(6.4)	10(1.2)	190(7.8)
Cerebral infarction, n(%)	556(58.0)	309(48.0)	176(21.1)	1041(42.7)
Spinal or osteoarticular disease, n(%)	102(10.6)	183(28.4)	488(58.4)	773(31.7)
Others, n(%)	162(16.9)	111(17.2)	161(19.3)	434(17.8)
**Hypertension, n(%)**	660(68.8)	356(55.3)	660(79.0)	1274(52.3)
**Diabetes, n(%)**	260(27.1)	116(18.0)	100(12.0)	476(19.5)
**Hyperlipidemia, n(%)**	61(6.4)	30(4.7)	40(4.8)	131(5.4)
**Heart disease, n(%)**	277(28.9)	106(16.5)	85(10.2)	468(19.2)

Civil servant^a^: Professional, commercial or technical employee of a state agency

Worker^b^: Service, production, transportation and some manual works

Farmer^c^: Agricultural, forestry, animal husbandry and fishery works

Others: workers not otherwise classified

**Fig. 3:**
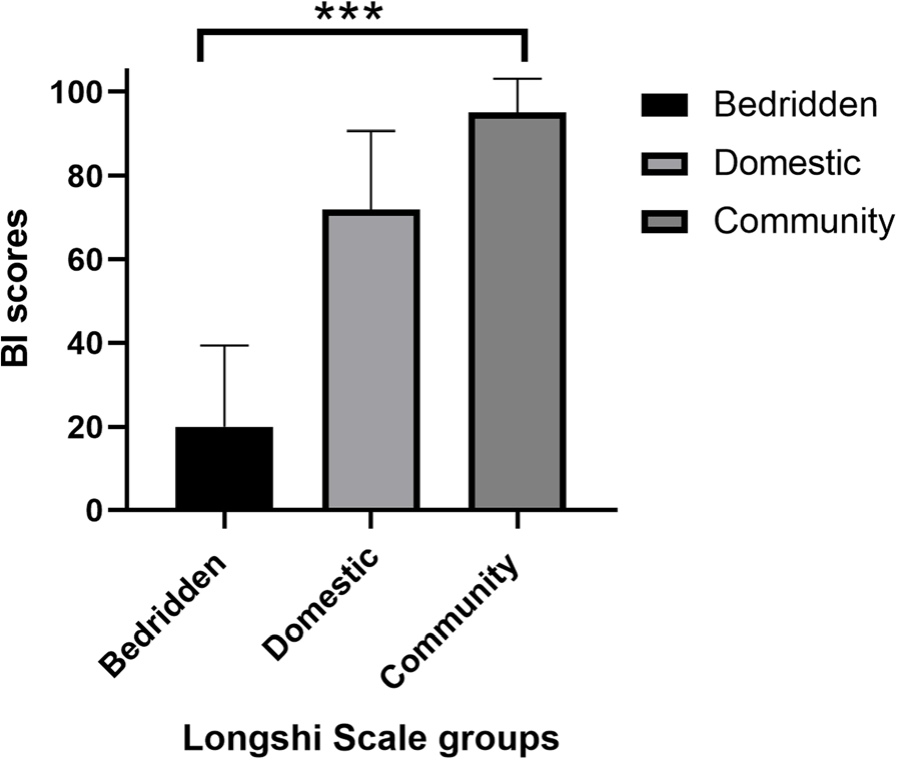
The differences of Barthel index scores based on groups of Longshi Scale

Figure 3 shown that there was a significant difference between the average BI scores of the Longshi Scale groups (*p* ≤ 0.001, Table 3). The patients in the community group had the highest mean BI total scores (95.01, SD = 8.16), while those of the bedridden group had the lowest (19.99, SD = 19.32).

**Table 3:** The differences of Barthel Index scores based on groups of Longshi Scale

Groups of longshi Scale	Barthel index scores (mean ± SD)	*F*-value	*P*-value
Bedridden	19.99 ± 19.32	5032.94	< 0.001
Domestic	71.86 ± 18.81		
Community	95.01 ± 8.16		

**Fig. 4:**
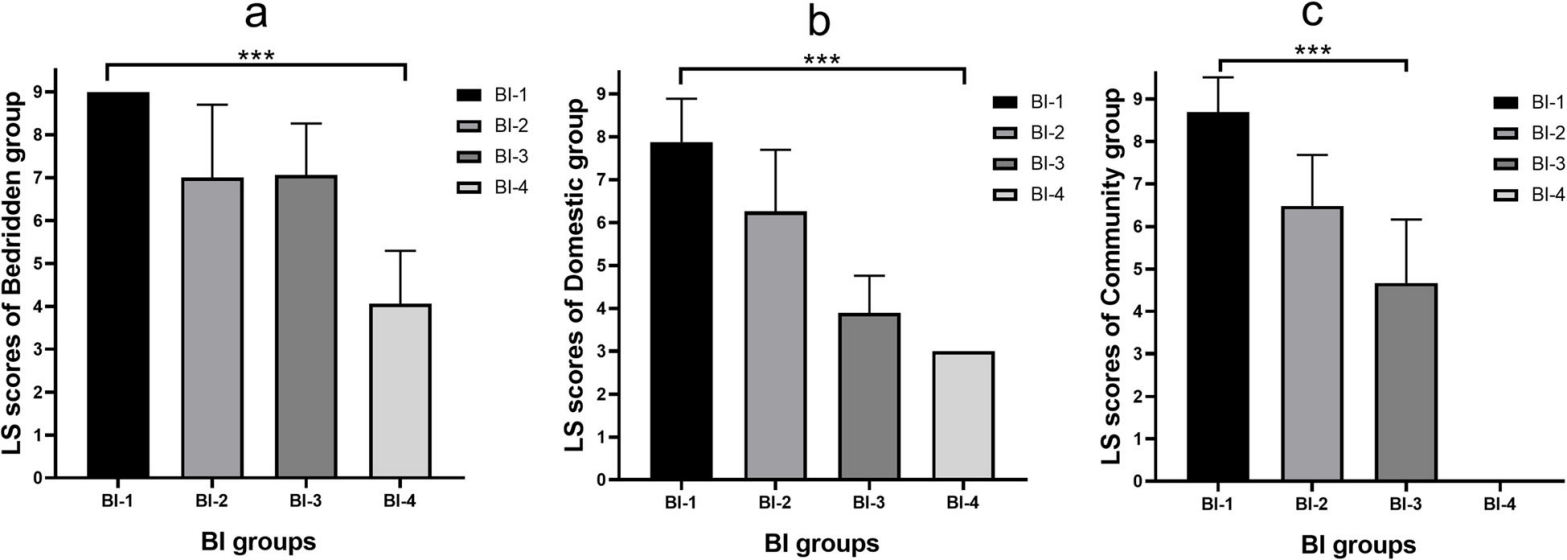
Differences of Longshi Scale scores in Bedridden group(4**a**), domestic group (4**b**) and community group(4**c**) based on groups of BI. BI-1: slight dependence, BI-2: moderate dependence, BI-3: severe dependence, BI-4: total dependence

In the Table 4, the Longshi Scale scores show significant differences between the BI groups (slight dependence, moderate dependence, severe dependence, and total dependence). The bedridden group had the lowest average score in the BI total dependence groups (*p* ≤ 0.001) (Fig. 4a). In the domestic group the highest mean Longshi Scale scores were seen in the BI’s slight dependence group (Fig. 4b). Significant differences in average Longshi Scale scores were also observed in the domestic group (*p* ≤ 0.001, Table 4). The average Longshi Scale scores in the community group ranged from severe to slight dependence (Fig. 4c). There was a significant difference among them in the community group (*p* ≤ 0.001).

**Table 4:** The differences of Longshi Scale scores based on the groups of Barthel index

Groups of Longshi Scale scores	Groups of Barthel index				*F*-value	*P*-value
	slight dependence (*n* = 681)	moderate dependence (*n* = 604)	severe dependence(*n* = 601)	total dependence (*n* = 552)		
Bedridden, n(%)	2(0.3)	19(3.1)	391(65.1)	547(99.1)		
Scores (mean ± SD)	9.00 ± 0.00	7.00 ± 1.70	7.06 ± 1.20	4.06 ± 1.24	465.90	< 0.001
Domestic, n(%))	89(13.1)	352(58.3)	198(32.9)	5(0.9)		
Scores (mean ± SD)	7.88 ± 1.01	6.26 ± 1.44	3.89 ± 0.88	3.00 ± 0.00	267.84	< 0.001
Community, n(%)	590(86.6)	233(38.6)	12(2.0)	0(0)		
Scores (mean ± SD)	8.69 ± 0.82	6.48 ± 1.20	4.67 ± 1.50	-	522.26	< 0.001

The Spearman correlation coefficients between the Longshi Scale scores and BI scores were 0.869 in the bedridden group, 0.848 domestic in the domestic and 0.828 in the community group. Those correlations were all significant at the *p* ≤ 0.001 level of confidence (Table 5).

**Table 5:** The correlations between Longshi Scale scores and Barthel Index scores

Groups of Longshi Scale	Longshi Scale scores (mean ± SD)	Barthel Index scores (mean ± SD)	correlation coefficient	*P*-value
Bedridden	5.35 ± 1.93	19.99 ± 19.32	0.869	< 0.001
Domestic	5.73 ± 1.84	71.86 ± 18.81	0.848	< 0.001
Community	8.02 ± 1.43	95.01 ± 8.16	0.828	< 0.001

## Discussion

These results suggest that the Longshi Scale’s pictorial approach scale of ADL in effectively distinguishes the ADL ability of older adults with a disability. The data also demonstrated that the Longshi Scale classifications correlated well with those using the BI. Both scales are useful in assessing the functional disability among the older adults.

The subjects in the Longshi Scale’s community group had the highest mean BI total scores (95.01, SD = 8.16), while those in its bedridden group had the lowest (19.99, SD = 19.32) indicating more severe loss of function. There was a significant difference in average BI scores among the three Longshi Scale groups (*p* ≤ 0.001, Table 3). These findings confirm that the Longshi Scale groups effectively discriminate among levels of ADL performance. That finding is consistent with those of previous researches [11, 20] that the ADL ability of older adults is positively related to their scope of their activities. The categorization and scoring system of the Longshi Scale make it easier to assess and understand elderly persons’ functional status.

The average scores of the three Longshi Scale groups showed significant differences among the 4 BI subgroups (slight, moderate, severe and total dependence) (*p* ≤ 0.001). The average scores of the three Longshi Scale groups gradually increased from the total dependence group to the slight dependence group. That shows that the Longshi Scale’s picture-based items can reflect the differences in ADL ability among older patients. Pictures might be superior to word-based scales with some older adults or very young patients [21–23].

The Spearman correlation coefficients relating the Longshi Scale and BI scores confirm a strong positive correlation with all three Longshi Scale groups. On that basis the Longshi Scale can be said to effectively reflect the ADL ability of older adult patients and to have useful clinical validity. The Longshi Scale offers a useful alternative in future research and in the clinic.

The assessment of community-dwelling older adults who may have an undiagnosed chronic illness or disability is of great importance [24, 25]. Indeed, such older adults may be at great risk of having a low level of self-care despite their community-dwelling status, which can have a profound effect on their quality of life [26, 27]. The Longshi Scale should be useful for evaluating their self-care ability and any need for intervention [28]. It can be used to assess older adults’ ability to maintain their own health, whether a serious health conditions exists or not.

Previous studies have shown that the Longshi Scale is a simple to use, yet reliable and scientific. It can be used with different groups of people and indeed by non-medical personnel or even patients themselves [16, 29]. With an aging society, it is necessary to establish a long-term care service system offering medical security system to the older adults [30]. The picture-based Longshi scale could be of great utility in this effort, especially for the elderly disabled persons with certain complications. Quick and effective assessment of ADL ability can help older adults enjoy the benefits of appropriate and effective care. It can also save on the cost of care and reduce the pressure on older adults’ care services[31, 32]. Its use should therefore improve rehabilitation treatment, guide the continuity of care and ensure adequate social support and disability benefits for the older adults[27].

There were some limitations in this study. Firstly, this was a cross-sectional cohort study. Its findings might usefully be extended by examining the changes in the Longshi Scale scores of the elderly disabled persons over time, with and without professional intervention. The scale’s sensitivity to change needs to be documented. Secondly, the data were collected by interviewing the patients or caregivers, which lead to information bias. However, based on a previous research, the results showed that Longshi Scale could potentially provide an efficient way to assess the ADL in disabled patients. Thirdly, the participants in this study were all-Chinese population, which was limited generalizability to other population.

Despite these limitations, this has been the first study to document conclusively the Longshi Scale’s ability to distinguish among levels of disability in elderly subjects. That despite its being a multicenter study using evaluators with different levels of training.

## Conclusions

The picture-based Longshi scale can effectively distinguish the levels of ADL ability in older adults. The scale shows great potential for improving the design of rehabilitation programs, improving the continuity of care, and adjudging appropriate disability benefits for the older adults.

## Data Availability

The datasets that support the findings of this study are available from the corresponding author (Yulong Wang) or the first author (Jingpu Zhao) upon reasonable request.

## Abbreviations

ADL: Activities of daily living
BI: Barthel Index
ICC: Intraclass Correlation Coefficient
MMSE: Mini-mental State Examination
CT: Computerized tomography
MRI: Magnetic resonance imaging
QRL: Quicker Recovery Line; SD:Standard deviation
SPSS: Statistical Package for the Social Sciences

## References

[1] National Economic. and Social Development Statistical Bulletin of the People’s Republic of China in 2019 http://www.stats.gov.cn/tjsj/zxfb/202002/t20200228_1728913.html.

[2] McPhee JS, French DP, Jackson D, Nazroo J, Pendleton N, Degens H (2016). Physical activity in older age: perspectives for healthy ageing and frailty. Biogerontology.

[3] Roberts CE, Phillips LH, Cooper CL, Gray S, Allan JL (2017). Effect of Different Types of Physical Activity on Activities of Daily Living in Older Adults: Systematic Review and Meta-Analysis. J Aging Phys Act.

[4] Groessl EJ, Kaplan RM, Rejeski WJ, Katula JA, Glynn NW, King AC, Anton SD, Walkup M, Lu CJ, Reid K (2019). Physical Activity and Performance Impact Long-term Quality of Life in Older Adults at Risk for Major Mobility Disability. Am J Prev Med.

[5] Phillips SM, Wójcicki TR, McAuley E (2013). Physical activity and quality of life in older adults: an 18-month panel analysis. Qual Life Res.

[6] Reed C, Belger M, Vellas B, Andrews JS, Argimon JM, Bruno G, Dodel R, Jones RW, Wimo A, Haro JM (2016). Identifying factors of activities of daily living important for cost and caregiver outcomes in Alzheimer’s disease. International psychogeriatrics.

[7] Fässberg MM, Cheung G, Canetto SS, Erlangsen A, Lapierre S, Lindner R, Draper B, Gallo JJ, Wong C, Wu J (2016). A systematic review of physical illness, functional disability, and suicidal behaviour among older adults. Aging Ment Health.

[8] Zeng Y, Feng Q, Hesketh T, Christensen K, Vaupel JW (2017). Survival, disabilities in activities of daily living, and physical and cognitive functioning among the oldest-old in China: a cohort study. Lancet.

[9] Connolly D, Garvey J, McKee G (2017). Factors associated with ADL/IADL disability in community dwelling older adults in the Irish longitudinal study on ageing (TILDA). Disabil Rehabil.

[10] Katz S, Ford AB, Moskowitz RW, Jackson BA, Jaffe MW. Studies of illness in the aged. The index of ADL: A standardized measure of biological and psychosocial function. Jama. 1963;185:914–9. 10.1001/jama.1963.03060120024016 14044222

[11] Elbaz A, Vicente-Vytopilova P, Tavernier B, Sabia S, Dumurgier J, Mazoyer B, Singh-Manoux A, Tzourio C (2013). Motor function in the elderly: evidence for the reserve hypothesis. Neurology.

[12] Collin C, Wade DT, Davies S, Horne V (1988). The Barthel ADL Index: a reliability study. Int Disabil Stud.

[13] Dickinson EJ (1992). Standard assessment scales for elderly people. Recommendations of the Royal College of Physicians of London and the British Geriatrics Society. J Epidemiol Community Health.

[14] Ribeiro D, Lenardt MH, Lourenço TM, Betiolli SE, Seima MD, Guimarães CA (2018). The use of the functional independence measure in elderly. Revista gaucha de enfermagem.

[15] Saito T, Izawa KP, Matsui N, Arai K, Ando M, Morimoto K, Fujita N, Takahashi Y, Kawazoe M, Watanabe S (2017). Comparison of the measurement properties of the Functional Independence and Difficulty Scale with the Barthel Index in community-dwelling elderly people in Japan. Aging clinical experimental research.

[16] Wang Y, Guo S, Zheng J, Wang QM, Zhang Y, Liang Z, Zhang L, Yang Y, Zhai H, Chen M (2019). User testing of the psychometric properties of pictorial-based disability assessment Longshi Scale by healthcare professionals and non-professionals: a Chinese study in Shenzhen. Clinical rehabilitation.

[17] Dumurgier J, Tzourio C (2020). Epidemiology of neurological diseases in older adults. Rev Neurol.

[18] Mahoney FI, Barthel DW. Functional evaluation: The Barthel Index. Maryland State Med J. 1965;14:61–5. 14258950

[19] Shah S, Vanclay F, Cooper B (1989). Improving the sensitivity of the Barthel Index for stroke rehabilitation. J Clin Epidemiol.

[20] Tanigawa T, Takechi H, Arai H, Yamada M, Nishiguchi S, Aoyama T (2014). Effect of physical activity on memory function in older adults with mild Alzheimer’s disease and mild cognitive impairment. Geriatr Gerontol Int.

[21] Ćwirlej-Sozańska AB, Sozański B, Wiśniowska-Szurlej A, Wilmowska-Pietruszyńska A (2018). An assessment of factors related to disability in ADL and IADL in elderly inhabitants of rural areas of south-eastern Poland. Annals of agricultural environmental medicine: AAEM.

[22] Vermeulen J, Neyens JC, van Rossum E, Spreeuwenberg MD, de Witte LP (2011). Predicting ADL disability in community-dwelling elderly people using physical frailty indicators: a systematic review. BMC Geriatr.

[23] Hopman-Rock M, van Hirtum H, de Vreede P, Freiberger E (2019). Activities of daily living in older community-dwelling persons: a systematic review of psychometric properties of instruments. Aging clinical experimental research.

[24] Wei K, Nyunt MS, Gao Q, Wee SL, Yap KB, Ng TP (2018). Association of Frailty and Malnutrition With Long-term Functional and Mortality Outcomes Among Community-Dwelling Older Adults: Results From the Singapore Longitudinal Aging Study 1. JAMA Netw Open.

[25] Chen W, Fukutomi E, Wada T, Ishimoto Y, Kimura Y, Kasahara Y, Sakamoto R, Okumiya K, Matsubayashi K (2013). Comprehensive geriatric functional analysis of elderly populations in four categories of the long-term care insurance system in a rural, depopulated and aging town in Japan. Geriatr Gerontol Int.

[26] Collaboration OAoST. Calculation of sample size for stroke trials assessing functional outcome: comparison of binary and ordinal approaches. Int J Stroke 2008;3(2):78–84. 10.1111/j.1747-4949.2008.00184.x 18705999

[27] Gobbens RJJ, van der Ploeg T (2020). The Prediction of Mortality by Disability Among Dutch Community-Dwelling Older People. Clin Interv Aging.

[28] Tierney SM, Woods SP, Weinborn M, Bucks RS (2018). Real-world implications of apathy among older adults: Independent associations with activities of daily living and quality of life. J Clin Exp Neuropsychol.

[29] Wang Y, Li S, Pan W, Xiao P, Zhang J, Wang QM, Luo X, Wang Y (2020). Evaluation of the disability assessment Longshi scale: A multicenter study. J Int Med Res.

[30] Chen S, Zheng J, Chen C, Xing Y, Cui Y, Ding Y, Li X (2018). Unmet needs of activities of daily living among a community-based sample of disabled elderly people in Eastern China: a cross-sectional study. BMC Geriatr.

[31] Ran L, Kong H, Du M, He J, Zhong Q, Ran Y, Si Y, Zhang J, Yao C, Luo H (2019). Comparison of health-related quality of life between the Han and Yi ethnicity elderly in the Yi autonomous areas of Yunnan Province. BMC Geriatr.

[32] Claesson L, Lindén T, Skoog I, Blomstrand C (2005). Cognitive impairment after stroke - impact on activities of daily living and costs of care for elderly people. The Göteborg 70 + Stroke Study. Cerebrovasc Dis.

